# Anatomical Variants of the Cervical Spine and Sports-Related Injury in Athletes: A Narrative Review

**DOI:** 10.3390/sports14080355

**Published:** 2026-08-18

**Authors:** Juan Alberto Sanchis-Gimeno, Alejandro Bruna-Mejías, Mathias Orellana-Donoso, Juan José Valenzuela-Fuenzalida, Gustavo Oyanedel-Amaro, José Eduardo León-Rojas

**Affiliations:** 1 Grupo de Investigación Anatomía Valencia (GIAVAL), Faculty of Medicine, University of Valencia, 46001 Valencia, Spain; juan.sanchis@uv.es; 2 Departamento de Ciencias y Geografía, Facultad de Ciencias Naturales y Exactas, Universidad de Playa Ancha, Valparaíso 2360072, Chile; alejandro.bruna@unab.cl; 3 Escuela de Medicina, Facultad de Medicina, Universidad Andrés Bello, Vina del Mar 2520000, Chile; 4 Escuela de Medicina, Universidad Finis Terrae, Santiago 7501015, Chile; mathor94@gmail.com; 5 Departamento de Morfología, Facultad de Medicina, Universidad Andrés Bello, Santiago 8370146, Chile; juan.kine.2015@gmail.com; 6 Departamento de Ciencias Química y Biológicas, Facultad de Ciencias de la Salud, Universidad Bernardo O’Higgins, Santiago 8370993, Chile; 7 Faculty of Health and Social Sciences, University of the Americas, Santiago 8370040, Chile; g.oyanedelamaro@gmail.com; 8 Cerebro, Emoción y Conducta (CEC) Research Group, Escuela de Medicina, Universidad de las Américas (UDLA), Quito 170521, Ecuador

**Keywords:** cervical spine, anatomical variants, athletes, sports injury, cervical stenosis, cervical cord neurapraxia, stingers, thoracic outlet syndrome, return to play, narrative review

## Abstract

Congenital and developmental cervical spine variants, as well as acquired or sport-adapted cervical morphologies, may alter canal reserve, segmental stability, foraminal dimensions, and the cervicothoracic outlet in athletes. This narrative review aimed to integrate the anatomically and methodologically heterogeneous literature linking these findings with sports-related injury and return-to-play decisions. PubMed/MEDLINE was searched from database inception through 6 July 2026 using controlled vocabulary and free-text terms for cervical anatomy, specific variants, athletic exposure, neurological syndromes, injury mechanisms, and return to sport; reference lists of relevant publications were also examined. English-language human studies were selected for direct anatomical, biomechanical, clinical, or athlete relevance and synthesized by evidence type, mechanism, vertebral level, and sport. The strongest athlete-specific evidence concerned developmental or functional cervical stenosis, space available for the cord, cervical cord neurapraxia, transient quadriparesis, and stingers in collision sports. Evidence for atlas arch defects, os odontoideum, Klippel–Feil syndrome, cervical spondylolysis, and cervical rib-related thoracic outlet syndrome was predominantly case-based or derived from expert guidance. These findings support cautious, individualized interpretation rather than universal clearance rules. Clinical assessment should integrate symptoms, neurological findings, imaging, dynamic stability, recurrence, and sport-specific exposure while recognizing that prospective athlete-specific evidence remains limited.

## 1. Introduction

The cervical spine functions as a mobile neural-protection system rather than simply as a column of seven vertebrae. During sport, it may be exposed to head impact, forced flexion or extension, axial compression, traction, shoulder depression, rapid rotation, repeated end-range loading, and forces transmitted through equipment or water [1,2,3,4,5,6]. These exposures help explain why morphology that is clinically silent in daily life may become relevant during a scrum, tackle, throw, dive, collision with boards, cheerleading basket toss, or overhead training cycle.

In this review, anatomical variant refers specifically to a congenital or developmental departure from typical cervical morphology, including developmental canal narrowing, atlas arch defects, os odontoideum, odontoid hypoplasia, atlantoaxial instability, Klippel–Feil segmentation defects, cervical ribs, elongated C7 transverse processes, and cervical spondylolysis. Acquired or sport-adapted findings—such as disc degeneration, osteophytosis, foraminal narrowing, altered alignment, and post-traumatic or postoperative morphology—were treated as a separate category of cervical morphology rather than as true congenital variants. Both categories were retained when they could plausibly modify canal reserve, stability, neural or vascular passageways, or load transfer in a sport-relevant context [7,8,9,10,11,12,13,14,15,16,17,18].

Historically, much of the sports literature has focused on catastrophic cervical injuries in American football, rugby, diving, ice hockey, and other collision or forceful-impact sports [1,2,3,4,5,6]. A second body of evidence centers on transient quadriparesis, cervical cord neurapraxia, and stenosis [7,8,9]. A third group of studies concerns stingers and burners, in which foraminal narrowing, canal geometry, and collision mechanics interact with brachial plexus and cervical root vulnerability [10,11,12]. In parallel, case reports describe less common but anatomically informative variants, including bipartite atlas in football, atlas fusion failure after sport injury, os odontoideum in boxing, C6 spondylolysis in judo or football, and cervical ribs in athletes with thoracic outlet syndrome [13,14,15,16,17,18]. However, these evidence streams remain fragmented: injury registries often describe sport, mechanism, and vertebral level without characterizing pre-existing anatomy, whereas variant-specific reports commonly provide detailed imaging but no exposed-population denominator [6,13,14,15,16,17,18]. This clinically important mismatch can encourage either over-restriction based on isolated case reports or under-recognition of a consequential but uncommon anatomical finding.

Accordingly, this narrative review addressed the following questions: Among athletes and sport participants, which congenital or developmental cervical variants and acquired or sport-adapted cervical morphologies have been reported? How might they modify cervical biomechanics? What injury, clinical, and return-to-play implications are supported by the available evidence? The review aimed to integrate these bodies of evidence by mechanism, vertebral level, and sport while distinguishing cohort- and review-level evidence from case reports and expert guidance. Its purpose was to support proportionate clinical reasoning, imaging interpretation, prevention, follow-up, and individualized return-to-play decision-making rather than to prescribe a universal eligibility rule.

## 2. Materials and Methods

### 2.1. Review Design and Question

This study was a clinically oriented narrative review. It addressed the questions stated in the Introduction and was reported with reference to established narrative-review guidance and the SANRA domains [19,20,21,22]. Because the available literature includes epidemiological studies, imaging studies, biomechanical analyses, registries, clinical reviews, case series, case reports, and return-to-play guidance with heterogeneous definitions and outcomes, the objective was critical integration rather than quantitative pooling.

### 2.2. Information Sources and Search Period

PubMed/MEDLINE was searched from database inception to 6 July 2026. No publication-date restriction was applied because foundational studies and historical policy papers were required to trace the development of screening and return-to-play practice. Searches combined the MeSH terms “Cervical Vertebrae,” “Athletes,” “Wounds and Injuries,” and “Return to Sport” with free-text terms covering the cervical region; specific variants and morphologies; athlete and sport exposure; neurological syndromes and injury patterns; imaging measures; and return to play. Search combinations were adapted iteratively to the anatomical finding, syndrome, or sport under review. Backward citation searching of selected reviews, cohorts, registries, and case reports was used to identify older or narrowly indexed publications.

English-language human publications were prioritized because the review team could reliably evaluate the nuanced anatomical, neurological, and return-to-play terminology used in those reports. This pragmatic restriction may have excluded relevant non-English evidence and is acknowledged as a potential source of language bias. General cervical-spine publications were retained only when required to interpret a finding with clear sports relevance.

### 2.3. Eligibility, Selection, and Synthesis

Eligible sources included athlete-specific clinical and epidemiological studies, imaging or morphometric studies, biomechanical analyses, cohort and registry studies, systematic or narrative reviews, consensus or return-to-play guidance, and case reports or small series describing rare variants. Sources were selected purposively for direct relevance to anatomy, biomechanics, injury mechanism, clinical assessment, or return to play. Non-human studies, non-cervical conditions, and reports without a clear cervical-anatomy or sport connection were excluded.

The evidence was synthesized in three complementary dimensions: (1) anatomical mechanism (canal reserve, stability, foraminal or root mechanics, adjacent-segment load transfer, and neurovascular outlet dimensions); (2) vertebral level from the craniovertebral junction to C7–T1; and (3) sport-specific exposure. Evidence type was preserved during interpretation: findings from systematic reviews, cohorts, registries, imaging studies, and biomechanical analyses were distinguished from case-based evidence and expert guidance. The synthesis focused on consistency, clinical applicability, uncertainty, and knowledge gaps; no pooled estimate or formal risk-of-bias score was calculated.

### 2.4. Evidence Interpretation and Terminology

To reduce overstatement, the manuscript uses association and possibility language that reflects the underlying study design. Cohort, registry, systematic-review, and athlete-imaging findings are identified as such, whereas isolated cases are presented as illustrative rather than generalizable. The review therefore does not treat the absence of reported cases as evidence of safety or a case report as proof of a universal contraindication.

Throughout the review, anatomical risk modifier is preferred to anatomical risk factor when causality has not been established. A reduced Torg–Pavlov ratio may indicate smaller radiographic canal dimensions, but its clinical meaning depends on magnetic resonance imaging (MRI), functional stenosis, space available for the cord (SAC), cord deformation, and clinical history [23,24,25]. Cervical ribs are more prevalent among patients with thoracic outlet syndrome (TOS) than among healthy populations, but the variant alone does not establish symptomatic disease; clinical relevance depends on coexisting symptoms, loading, and demonstrable compression [26,27,28].

## 3. Results

### 3.1. Anatomical and Biomechanical Framework

#### 3.1.1. The Cervical Spine as an Integrated Kinetic and Neural-Protection System

Cervical injury consequences are shaped by the interaction between external load, segmental alignment, motion constraints, soft-tissue stabilizers, canal dimensions, foraminal geometry, and neurovascular tunnels. The upper cervical spine supports the skull and provides substantial rotation; the subaxial spine distributes flexion, extension, lateral bending, and coupled rotation; and the cervicothoracic junction transfers load between the mobile neck and the relatively rigid thorax. A variant or acquired morphology at one level may redistribute load to adjacent segments, particularly when collision forces occur faster than protective muscular responses [1,5].

Four principles recur across the literature. First, reduced canal reserve or SAC may increase the likelihood of transient cord deformation during hyperflexion, hyperextension, or axial loading [8,9]. Second, odontoid abnormalities, ligamentous laxity, or segmentation defects may compromise dynamic stability. Third, extension, rotation, lateral bending, and axial load can narrow the neural foramen or tension the upper brachial plexus, contributing to stingers [10,12]. Fourth, a cervical rib or elongated C7 transverse process may reduce outlet dimensions that become clinically relevant during repetitive overhead motion, shoulder-girdle hypertrophy, or loaded posture [26,27]. These principles organize the results but do not constitute a validated prediction model.

#### 3.1.2. Developmental and Functional Cervical Canal Stenosis

Developmental cervical stenosis is the most extensively studied anatomical modifier in athletes with transient quadriparesis or cervical cord neurapraxia. Torg et al. described a transient post-traumatic neurological syndrome ranging from paresthesia to paresis or plegia and noted smaller canal-to-body ratios in many affected athletes [9]. Pavlov et al. formalized the sagittal canal-to-vertebral-body ratio on lateral radiographs, subsequently termed the Torg–Pavlov ratio [23].

The ratio remains an imperfect screening tool because it is a two-dimensional surrogate affected by projection, body habitus, vertebral level, and bone dimensions rather than the actual cord–CSF–disc–ligament relationship. Professional and rookie football cohorts contained many asymptomatic athletes with reduced ratios, which limited specificity [29,30]. MRI-based assessment of SAC, cerebrospinal-fluid buffering, cord deformation, and functional stenosis is therefore more clinically informative than the ratio alone [8,24].

The clinically important distinction is between developmental narrowing as an imaging morphology and functional stenosis as a dynamic pathophysiological state. Functional stenosis may reflect congenital narrowing together with disc protrusion, ligamentous infolding, alignment, or post-traumatic or degenerative change. A history of recurrent bilateral symptoms, cervical cord neurapraxia, or cord signal abnormality warrants greater concern than an isolated reduced ratio in an asymptomatic athlete [31,32,33].

#### 3.1.3. Foraminal Morphology, Stingers, and Cervical Roots

Stingers and burners are common transient neurological injuries in collision athletes. Proposed mechanisms include brachial-plexus traction, a direct supraclavicular blow, and cervical root compression during extension, rotation, or lateral bending. Relevant anatomy therefore includes foraminal dimensions, disc height, uncovertebral and facet morphology, osteophytes, and root course [10,11].

Meyer et al. reported approximately a threefold stinger risk among collegiate football players with a Torg ratio below 0.8 and more complicated courses in those with stenosis [12]. Castro et al., however, found that initial incidence varied with position and body type and observed a mean ratio of 0.924, cautioning against ratio-only prediction [34]. Together, these athlete cohorts suggest that canal and foraminal dimensions interact with position, technique, body habitus, neck strength, and cumulative exposure.

Reviews and expert guidance treat recurrent, bilateral, prolonged, or progressive episodes as more concerning than an isolated unilateral event. Such presentations warrant evaluation for central or foraminal stenosis, disc herniation, instability, or brachial plexus injury. Return is generally considered only after symptom resolution, full painless motion, normal strength and neurological examination, and appropriate imaging when episodes are recurrent or atypical [10,11,35].

#### 3.1.4. Congenital Fusion and Segmentation Variants

Congenital fusion, block vertebrae, and Klippel–Feil syndrome (KFS) alter the distribution of cervical motion. A fused segment has reduced local motion, while adjacent segments may experience compensatory motion, stress, or degeneration [36,37]. In collision athletes, this may concentrate flexion-extension, rotation, or axial load above or below the fusion. Expert guidance generally regards multilevel fusion, upper cervical involvement, instability, stenosis, and neurological symptoms as features of greater concern than an isolated, asymptomatic, stable single-level fusion [36,37].

Pattern-specific assessment is more informative than the diagnostic label alone. A lower cervical fusion without instability, stenosis, or neurological symptoms differs from upper cervical or occipitocervical fusion, basilar invagination, atlantoaxial instability, or an associated C1 arch anomaly. A published case of congenital posterior C1 arch hypoplasia, cervical fusion, atlantoaxial subluxation, and temporary quadriplegia illustrates the potential consequence of combined abnormalities but cannot define risk for all athletes with KFS [38].

#### 3.1.5. Cervical Ribs and the Cervicothoracic Outlet

Although cervical ribs and elongated C7 transverse processes arise from cervical anatomy, they usually present through upper-limb neurovascular symptoms rather than cord symptoms. A meta-analysis of 141 studies estimated cervical-rib prevalence at 29.5% among patients with TOS and 1.1% among healthy individuals [27]. In athletes, a reduced outlet may interact with repetitive abduction-external rotation, clavicular motion, scalene tension, muscle hypertrophy, and heavy cumulative training load.

Sports-related TOS includes neurogenic, venous, and arterial phenotypes with different urgency. Athlete cohorts and systematic reviews indicate that return to competition may be possible after accurate diagnosis and appropriate decompression, whereas persistent vascular compression or thrombosis requires a different risk response from positional paresthesia [26,39,40]. The athlete-specific literature also includes a case involving bilateral cervical ribs and a cohort examining predisposing anatomy after supraclavicular decompression [14,28]. Table 1 provides a concise evidence map and explicitly identifies the evidence basis for each anatomical finding.

### 3.2. Vertebra-by-Vertebra Synthesis

#### 3.2.1. Craniovertebral Junction and Occiput-C1

The craniovertebral junction (CVJ) comprises the occipital condyles, foramen magnum, atlas, axis, odontoid, and major stabilizing ligaments. Because it supports substantial rotation and contains the transition from brainstem to cervical cord, instability or neural compression can have serious consequences during collision, combat, gymnastics, diving, or trampoline mechanisms. Athlete-specific data are limited, so interpretation depends primarily on symptoms, neurological findings, and dynamic stability.

In athletes with Down syndrome, ligamentous laxity and C1–C2 morphology are recurring concerns. Reported radiographic prevalence varies, and plain radiographs do not reliably predict neurological events [46,52,53]. Contemporary guidance therefore emphasizes neurological symptoms and specialist assessment, particularly before activities involving repeated flexion-extension, tumbling, diving, contact, or falls [47,53].

Basilar invagination, atlas occipitalization, Chiari malformation, and congenital occipitocervical fusion are rarely studied in athletes. In the absence of prospective sport-specific evidence, cord or brainstem compression, syrinx, neurological symptoms, and demonstrated instability should be treated as potentially serious findings requiring individualized specialist review rather than as evidence for a universal rule.

#### 3.2.2. C1 Atlas

The atlas is a ring, and congenital anterior or posterior arch defects and a bipartite atlas can resemble acute fracture after sports trauma. Petraglia et al. described a collegiate football player with a bipartite atlas after a tackle; after CT and MRI assessment, the stable anomaly was not treated as an automatic contraindication [15]. Petre et al. described anterior and posterior atlas fusion failure after athletic injury and emphasized distinguishing congenital nonunion from acute fracture [16]. These case reports illustrate diagnostic problems but do not establish general clearance criteria.

The clinical significance of a C1 arch defect depends on symptoms, canal compromise, associated anomalies, and stability. A small midline cleft may be incidental, whereas a mobile fragment, combined ring defect, or atlantoaxial subluxation may threaten the cord. One case of posterior arch deformity with atlantoaxial subluxation and temporary quadriplegia illustrates the severe end of this spectrum [38]. Computed tomography (CT) is useful for cortical morphology; MRI evaluates the cord, ligaments, and edema; and dynamic imaging may be considered only after acute fracture or major ligament injury has been excluded.

Accordingly, clinicians should avoid both dismissing post-traumatic symptoms because a defect is congenital and treating a stable congenital cleft as an unstable acute fracture.

#### 3.2.3. C2 Axis and Odontoid Complex

The C2 vertebra is the pivot for much of cervical rotation, and odontoid and transverse-ligament integrity are central to atlantoaxial stability. Os odontoideum, odontoid hypoplasia, persistent ossicles, and nonunion may remain asymptomatic until trauma produces translation or cord compromise. Evidence in athletes is largely case-based.

Place and Enzenauer reported a case involving a boxer with cervical injury and os odontoideum [17]. Clinical guidance recommends CT, MRI, and, when safe, dynamic radiography to assess stability and neural compromise [44]. In an athlete, the relevant questions are whether the lesion is stable and whether myelopathy, cord signal change, recurrent symptoms, or excessive motion is present. An unstable or symptomatic os odontoideum should prompt specialist assessment; this recommendation is based on clinical guidance rather than athlete-specific prospective trials.

C1–C2 instability and odontoid abnormalities may coexist with ligamentous laxity in Down syndrome. Symptom-driven, risk-stratified assessment is favored over reliance on screening radiographs alone, with particular caution for contact, collision, gymnastics, diving, trampoline, and equestrian activities when symptoms or instability are present [46,53].

#### 3.2.4. C2–C3 and Multilevel Segmentation Patterns

C2–C3 fusion is a common KFS or block-vertebra pattern. Reduced motion at the fused level may be accompanied by compensatory adjacent-segment motion and degeneration [36,37]. In athletes, interpretation should encompass upper cervical stability, canal dimensions, associated anomalies, adjacent-segment disease, and the specific load profile of the sport.

Expert return-to-play reviews distinguish a stable, asymptomatic single-level fusion from multilevel or upper cervical fusion, stenosis, instability, and neurological symptoms [36,43,49]. Because evidence is mainly observational or expert-based, the diagnosis alone should not determine clearance.

#### 3.2.5. C3–C4 and C4–C5

C3–C5 contributes to global subaxial cord reserve and is usually considered within multilevel developmental or functional stenosis rather than as an isolated variant. Dynamic compression may occur at a level different from the site of impact because disc, ligament, and posterior-element relationships change during flexion and extension [8,9,23,24,25]. Pediatric and adolescent patterns also differ because of skeletal maturity, ligamentous laxity, head-to-body ratio, and motion fulcrum; however, youth injury series usually report injury level rather than pre-existing morphology [54,55]. Consequently, current evidence cannot estimate level-specific risk for isolated C3–C4 or C4–C5 variants in athletes.

#### 3.2.6. C5–C6

C5–C6 is a mobile subaxial segment frequently involved in disc degeneration, foraminal narrowing, and radicular symptoms in sports populations [5,56,57,58]. In a water-sport series, flexion and axial loading were common and C5 fracture was prominent [56]. Rugby cohorts described career-related disc and foraminal changes [57,58]. These acquired or sport-adapted morphologies should be distinguished from congenital variants, although both may reduce neurological reserve.

#### 3.2.7. C6

C6 spondylolysis is rare, and athlete evidence consists mainly of case reports. Sasa et al. described a 12-year-old judo athlete with bilateral C6 spondylolysis, grade I spondylolisthesis, and mild C6–C7 disc degeneration; finite-element analysis indicated increased disc stress and motion, particularly during axial rotation [18]. This report may be particularly informative because it linked clinical imaging with biomechanical modeling, but it cannot define population risk.

Amin et al. described bilateral C6 spondylolysis and spondylolisthesis in an adolescent baseball player, and Alton et al. described pediatric C6 defects in relation to American football [13,50]. These cases suggest that symptoms and management vary with instability, neurological findings, healing, and sport demands; they do not support sport-specific prevalence estimates.

#### 3.2.8. C6–C7 and C7–T1

The C6–C7 segment is a frequent site of lower cervical disc and foraminal pathology. A case involving a professional rugby league player described stenosis and C7 radiculopathy after repeated trauma [59]. At this level, extension-compression and foraminal narrowing may affect exiting roots and contribute to stinger or radicular presentations.

C7 is the level from which cervical ribs and elongated transverse processes arise. These variants can contribute to neurovascular outlet narrowing during throwing, swimming, rowing, climbing, strength training, or sustained endurance posture. Cervical ribs have substantially greater prevalence in TOS populations than in healthy populations [27], and athlete reports describe neurogenic and vascular presentations [14,28,40].

Acute C7 fractures and posterior-element injuries can also produce transient or catastrophic neurological events without a pre-existing variant, as illustrated by a youth-football case [60]. Thus, variants modify the context and consequences of injury but do not replace the importance of force, mechanism, and acute structural damage (Table 2).

### 3.3. Sport-Specific Injury Synthesis

#### 3.3.1. American Football

American football accounts for much of the athlete-specific literature on cervical stenosis, transient quadriparesis, cervical cord neurapraxia, and stingers. Registry studies established axial loading and head-down contact as major mechanisms of catastrophic injury [6,61]. The sport combines large body mass, speed, protective equipment, tackling, blocking, and recurrent exposure, making technique and neurological reserve clinically relevant.

Studies have repeatedly described developmental narrowing among football players with transient quadriparesis or cervical cord neurapraxia, although causal certainty varies. Torg et al. characterized the syndrome and ratio, and Ladd and Scranton described congenital stenosis in affected athletes [9,25,62]. A single resolved episode does not by itself establish future catastrophic risk; recurrent episodes, persistent symptoms, functional stenosis, instability, and cord signal abnormality are more concerning.

Stingers are particularly common in football and may result from traction, direct blow, or foraminal/root compression. Athlete reviews describe their prevalence and management, while prospective and cohort data identify stenosis, foraminal narrowing, position, body type, and recurrence as relevant modifiers [10,11,12,34].

Football also provides case literature on bipartite atlas, C1 fusion failure, pediatric C6 spondylolysis, and C7 fracture with transient tetraplegia [13,15,16,60]. These reports illustrate why evaluation should define the level, morphology, stability, neurological status, and evidence basis rather than treating all neck injuries or incidental variants as equivalent.

#### 3.3.2. Rugby Union and Rugby League

Rugby has a distinct cervical profile because scrums, tackles, rucks, mauls, and unhelmeted collisions produce repeated loading. Reviews and registry studies describe position-dependent catastrophic injury, and French surveillance documented declining incidence during a period of prevention initiatives and scrum law changes [4,63,64].

Front-row players have also been studied for chronic disc, foraminal, and canal changes. Athlete cohorts reported age- or career-related degeneration and alternative radiographic measures of stenosis [57,58,65]. These findings suggest that rugby involves both acute mechanisms and acquired or sport-adapted morphology that may reduce canal or foraminal reserve.

Stingers occur in rugby as well as American football. A study of young rugby players supports the relevance of root and brachial-plexus mechanisms [66], and a professional rugby league case described stenosis and radiculopathy after repetitive trauma [59]. Return-to-play decisions should integrate the acute mechanism, position, acquired morphology, stenosis, neurological history, and recurrence.

#### 3.3.3. Ice Hockey

Ice hockey exposes the cervical spine to rapid impacts against boards, ice, other players, and equipment. Reviews emphasize axial loading, head-first contact, equipment-related immobilization challenges, and prevention [67,68].

Variant-specific evidence is less developed than in football. A professional-player case described spinal cord concussion without fixed canal compromise, showing that transient symptoms may occur in the absence of stenosis [69]. Conversely, congenital stenosis, recurrent neurapraxia, or cord symptoms still warrant MRI-based functional assessment.

Helmet and shoulder-pad configurations can alter cervical alignment during emergency immobilization [70,71]. When stenosis or an anatomical variant is suspected, secondary movement should be minimized and equipment management should follow sport-specific emergency protocols.

#### 3.3.4. Wrestling, Judo, Martial Arts, and Boxing

Combat and grappling sports expose the neck to flexion, extension, rotation, compression, traction, throws, and opponent-applied forces. The judo case report combined clinical imaging, finite-element modeling, and sport-specific advice, making it a useful mechanistic illustration rather than a generalizable risk estimate [18].

Wrestling literature includes a case of central cord syndrome in which congenital or degenerative narrowing may have been relevant [72]. The boxer case involving os odontoideum raises a different concern: repeated head impact and rotation may be consequential when upper cervical instability is present [17].

Evidence for clearance in combat sports remains largely case-based or expert-derived. Stable incidental findings may be compatible with participation after specialist clinical and imaging assessment [15,36,49]. Unstable os odontoideum, symptomatic KFS, C1–C2 instability, recurrent cord neurapraxia, and cord signal change represent substantially different situations and generally argue against contact exposure until specialist evaluation and treatment are complete [17,32,35,36,37,38,41,42,43,44,49]. These statements should be interpreted as expert guidance rather than validated sport-specific thresholds.

#### 3.3.5. Diving and Water Sports

Diving and water sports produce classic axial-loading injuries when the head strikes the bottom, a wave, or another object. Good and Nickel described a retrospective series in which flexion and axial loading were common and C5 fractures were prominent [56]. These injuries are primarily mechanism-driven, although pre-existing stenosis may reduce neurological reserve.

Water-sport series rarely characterize pre-existing cervical variants, which limits inference. Prevention therefore remains mainly environmental and behavioral. Athletes with known stenosis, fusion, instability, or previous neurapraxia should receive individualized counseling about head-first entry and forceful water impact.

#### 3.3.6. Gymnastics, Cheerleading, Trampoline, and Acrobatic Sports

Gymnastics, cheerleading, and trampoline expose the neck to falls, inversion, tumbling, forced flexion-extension, landing errors, and axial load. Catastrophic cheerleading reports include cervical fractures and ligament injuries, and later surveillance reported fewer catastrophic basket-toss injuries after the 2006–2007 rule change [73,74].

Pre-existing variants are rarely documented in these reports. Nevertheless, upper cervical instability, congenital fusion, functional stenosis, or cord signal abnormality may be clinically important in sports involving repeated tumbling, bridging, flips, and falls. This is especially relevant when Down syndrome-related C1–C2 instability is suspected [47,53].

#### 3.3.7. Baseball, Softball, Throwing Sports, and Overhead Athletes

Overhead athletes are less exposed to recurrent axial collision than football or rugby athletes, but they are central to the TOS literature [1,4,5,26,28,39,64]. Throwing, swimming, volleyball, tennis, rowing, and similar sports combine repeated shoulder elevation, scapular motion, clavicular rotation, scalene or pectoralis minor tension, and large cumulative training loads. These factors may convert a previously asymptomatic outlet predisposition into neurogenic or vascular symptoms [26,28,39]. Cervical ribs and elongated transverse processes can reduce baseline outlet dimensions, but they are neither necessary nor sufficient for TOS.

Baseball also appears in the cervical spondylolysis literature. Amin et al. described bilateral C6 spondylolysis and spondylolisthesis in an adolescent baseball player with neck pain and intermittent upper-limb symptoms [50]. Throwing athletes with neck pain, radiculopathy, or exertional upper-limb symptoms should be assessed for both lower cervical root disease and TOS rather than assuming a primary shoulder disorder [26,28,39,50].

#### 3.3.8. Endurance and Strength Sports

Swimming, rowing, weightlifting, climbing, and running can expose the cervicothoracic region to repeated shoulder elevation, scapular-control demands, cervical rotation or extension, hypertrophy, load carriage, and sustained posture. These sports are especially relevant to TOS because symptoms may be positional, exertional, neurogenic, or vascular; anatomical contributors include cervical ribs and anomalous bands [51].

A case involving a young female athlete with bilateral cervical ribs illustrates presentation outside collision sport [14]. Athlete cohorts indicate that return after TOS surgery may be possible, but outcome depends on neurogenic versus vascular phenotype, severity, decompression, and associated pathology [28,40]. Rare runner reports further illustrate the interaction between posture, repetitive arm motion, and anatomical predisposition [75].

#### 3.3.9. Cycling, Equestrian Sports, Skiing, Snowboarding, and Motorsports

Cycling, equestrian sports, skiing, snowboarding, and motorsports primarily produce cervical injury through falls, collisions, and rapid deceleration. In a nationwide US emergency-department study, cervical fracture patterns varied by age, sex, and activity; incidence increased by 35% from 2000 to 2015, driven largely by cycling-related injuries, with cycling prominent in males and horseback riding in females [76]. Evidence specifically characterizing pre-existing variants in these sports remains sparse.

When a fracture or spinal cord injury occurs, pre-existing stenosis, congenital fusion, instability, and postoperative anatomy should nonetheless be documented because they may influence neurological consequences, surgical planning, and return-to-play counseling [8,9,25,31,32,36,37,48,49]. These findings may modify management even when they did not cause the fall or crash.

#### 3.3.10. Basketball, Soccer, Lacrosse, and Other Field or Court Sports

Field and court sports produce fewer catastrophic cervical injuries than collision football or rugby but still involve falls, player-to-player contact, headers, awkward landings, and rapid impacts. An adolescent case series included spinal cord neurapraxia in football, ice hockey, and basketball, confirming that transient cord symptoms are not confined to traditional collision sports [77].

In high-school surveillance, football, wrestling, and girls’ gymnastics had the greatest cervical injury rates, although injuries occurred across many sports [78]. In basketball, soccer, lacrosse, and similar activities, an anatomical finding becomes most relevant when recurrent neurological symptoms, previous injury, stenosis, instability, or altered post-traumatic management is present.

#### 3.3.11. Adaptive Sports and Special Olympics

Among adaptive-sport contexts, Down syndrome-associated CVJ abnormalities and atlantoaxial instability are the best-described cervical concerns [46,47,53]. Routine radiographic screening of asymptomatic athletes remains controversial because radiographic abnormalities do not reliably predict neurological compromise [46,52,53]. Neck pain, weakness, sensory change, gait disturbance, persistent head tilt, hyperreflexia, or other myelopathic signs should prompt neurological and specialist assessment before activities involving substantial cervical exposure.

Historical Special Olympics policies reflected concern about cervical flexion-extension, direct neck pressure, diving, gymnastics, high jump, equestrian events, and selected contact sports in athletes with suspected C1–C2 instability [45,47]. Goldberg’s 1993 paper was retained only to document this historical policy context; contemporary interpretation relies on later literature supporting symptom-based, individualized assessment rather than automatic exclusion based on Down syndrome or an isolated radiographic finding [45,52,53]. Athletes with suspected symptomatic instability or cord compression require specialist evaluation before clearance (Table 3).

## 4. Clinical Implications for Evaluation and Return to Play

### 4.1. Practical Evaluation Pathway

A practical evaluation pathway begins with the athlete rather than the image. The meaning of a finding depends on symptoms, neurological examination, sport, position, recurrence, mechanism, and imaging context. An incidental cervical rib in an asymptomatic runner differs from arterial TOS in a pitcher; a stable bipartite atlas differs from an unstable C1 ring defect; and a reduced Torg–Pavlov ratio differs from MRI-confirmed functional stenosis after transient quadriparesis.

Red flags for immediate specialist assessment include bilateral neurological symptoms, symptoms involving more than one limb, transient quadriparesis or tetraparesis, persistent weakness, recurrent stingers, objective radiculopathy, myelopathic signs, gait disturbance, and bowel or bladder symptoms. Structural red flags include cord signal abnormality, canal compromise, fracture, dislocation, ligamentous disruption, and dynamic instability. Vascular TOS and symptoms provoked by minor trauma also require prompt specialist review.

Imaging should be driven by mechanism and symptoms. CT is particularly useful for atlas arch defects, osseous variants, fractures, pars or pedicle defects, and cervical ribs. MRI is required to evaluate cord signal, disc herniation, ligament injury, epidural pathology, functional stenosis, foraminal compromise, and soft tissue. Flexion-extension or other dynamic imaging may be considered when instability is suspected, but not acutely before fracture and major ligament injury have been excluded. Electrodiagnostic testing may assist when stinger, brachial plexopathy, or radiculopathy persists.

For sports physicians, preparticipation evaluation should be history- and examination-led rather than based on universal imaging. Advanced imaging is appropriate when symptoms, recurrent events, abnormal neurological findings, previous cervical surgery, or a potentially consequential variant changes the risk profile. Complex clearance decisions should involve sports medicine, spine surgery or neurosurgery, radiology, rehabilitation, and vascular expertise as indicated. Athletes who return should receive education about recurrence, technique or load modification, and symptom-triggered follow-up [35,36,49,81].

### 4.2. Integrated Return-to-Play Decision Framework

Return-to-play evidence remains limited and is dominated by reviews, expert guidance, and selected cohorts. Common criteria include being pain-free and neurologically normal, with full painless cervical motion, normal strength, no instability, no ongoing cord compression, and no progressive deficit [35,49,81]. For athletes with stenosis or previous cervical cord neurapraxia, recurrence, cord signal change, functional stenosis, or instability weighs strongly against collision exposure [31,32,33]. Table 4 standardizes the anatomical finding, principal risk, required investigations, absolute or near-absolute contraindications, relative concerns, and possible return criteria. Because prospective validation is lacking, the contraindication categories reflect convergent expert guidance rather than universal rules.

## 5. Discussion

Sports clinicians face a fragmented evidence base: population and registry studies characterize mechanisms and injury patterns but often omit pre-existing cervical anatomy, whereas detailed reports of rare variants are usually case-based and lack denominators. This gap is clinically important because the same imaging finding can lead to inappropriate reassurance or unnecessary exclusion when evidence strength, dynamic stability, and sport exposure are not considered together. The rationale for this review was therefore to integrate anatomy, biomechanics, evidence type, vertebral level, sport mechanism, and return-to-play implications within a single clinically proportionate framework.

The synthesis supports a four-layer model of cervical anatomical risk: structural geometry, dynamic biomechanics, tissue state, and exposure. Structural geometry includes canal and foraminal dimensions, odontoid and atlas integrity, segmentation pattern, and cervical-rib anatomy; dynamic biomechanics includes motion, stability, axial load, extension-compression, rotation, and traction; tissue state includes disc, ligament, cord, and neurovascular findings; and exposure includes sport, position, competition level, recurrence, cumulative load, equipment, and rules. Adverse outcomes are most plausible when several layers interact unfavorably. The model is interpretive and has not been validated as a prediction rule.

The strongest athlete-specific evidence concerns cervical stenosis and transient neurological events in collision sports [8,9,25,31,32,33,41,42,43]. The Torg–Pavlov ratio provided a practical historical measure [9,23], but reduced ratios are common in asymptomatic football cohorts and therefore have limited specificity [24,29,30]. Current interpretation gives greater weight to MRI-defined functional stenosis, SAC, cerebrospinal-fluid buffering, cord deformation or signal abnormality, and the athlete’s symptom history [8,24,31,32,33,41,42,43]. Canal dimensions matter, but the ratio should not be used alone for screening or clearance.

Level-specific synthesis indicates different clinical priorities. C1–C2 findings are infrequent but potentially serious because they may affect ring integrity, odontoid stability, or the cord [15,16,17,38,44,46,47]. C3–C7 morphology contributes to cord reserve [8,9,23,24,25]; C4–C7 disc and foraminal changes contribute to root or plexus presentations [10,11,12,34,57,58,59]; C6 spondylolysis offers only case-based mechanistic evidence [13,18,50]; and C7 variants extend assessment to neurogenic and vascular TOS [14,26,27,28,39,40,51,75]. These categories should not be collapsed into a single label of “cervical abnormality.”

Sport-specific mechanisms further modify interpretation. Football and rugby emphasize collision and axial load [1,4,5,6,61,63,64,79]; hockey adds board impact and equipment considerations [67,68,70,71]; combat and grappling sports add rotation and opponent-applied forces [18,72]; diving adds head-first environmental impact [3,56]; acrobatic sports add inversion and falls [73,74]; and overhead sports emphasize the neurovascular outlet [26,28,39,40,51]. Cycling and equestrian sports are prominent in cervical-fracture epidemiology [76]. A finding should therefore be interpreted against actual exposure, symptoms, neurological status, and dynamic stability rather than through a universal prohibition [35,36,49,81].

The principal evidentiary limitation is the mismatch between registries that lack detailed anatomy and case reports that lack denominators [6,13,14,15,16,17,18,38,50,61,63,73,74,76,78,79]. As a result, causal inference and variant-specific incidence or relative-risk estimates are possible for only a small subset of findings. The review’s cautious language and evidence labels are intended to prevent case-based observations from being read as population-level rules.

### 5.1. Research Priorities

Future work should move from static labels to prospective dynamic phenotyping. Contact-sport studies should integrate MRI canal and SAC metrics, foraminal dimensions, alignment, neck strength, position, exposure, injury history, and recurrent or subclinical neurological symptoms rather than focusing only on catastrophic injury.

Rare C1–C2 variants require multicenter registries capable of recording sport, symptoms, neurological findings, CT and MRI morphology, dynamic stability, treatment, recurrence, and return-to-play outcomes. Without denominators and longitudinal follow-up, clinicians will continue to rely on isolated cases and expert opinion.

TOS studies should report cervical ribs, elongated C7 transverse processes, scalene and pectoralis-minor anatomy, sport and competition level, phenotype, surgical approach, return timing, and recurrence. Athletes have distinctive performance demands, yet anatomical reporting remains variable [26,28].

Pediatric and adolescent research should treat growth, skeletal maturity, and sport specialization as modifiers. A young athlete with C6 spondylolysis, Down syndrome-related instability, or congenital stenosis differs from an adult collision athlete with acquired foraminal disease. Age-specific guidance is needed for safe and equitable participation.

### 5.2. Limitations

This narrative review used purposive rather than exhaustive identification, relied principally on PubMed/MEDLINE with backward citation searching, and was not prospectively registered. The English-language restriction may have introduced language bias, and no formal risk-of-bias scoring or meta-analysis was performed. Although the scope, search period, search domains, eligibility boundaries, synthesis approach, and evidence categories are reported, selection and interpretation bias remain possible [19,20,21,22].

The underlying literature is also heterogeneous in definitions, imaging thresholds, exposure denominators, and return-to-play outcomes. Prospective athlete-specific studies are few, registries seldom characterize pre-existing anatomy, and case reports cannot estimate incidence. Publication of unusual or dramatic cases may exaggerate perceived risk, while the absence of published cases does not establish safety. Consequently, most recommendations for rare variants remain based on case reports, small series, or expert guidance rather than validated prognostic data.

## 6. Conclusions

This review aimed to integrate evidence on congenital or developmental cervical variants and acquired or sport-adapted morphologies in athletes. The synthesis indicates that the strongest athlete-specific evidence concerns developmental or functional stenosis, cervical cord neurapraxia, transient quadriparesis, and stingers in collision sports. Evidence for atlas arch defects, os odontoideum, KFS, C6 spondylolysis, and cervical rib-related TOS is predominantly case-based or expert-derived and therefore supports cautious interpretation rather than universal clearance rules.

The main clinical conclusion is that anatomy should be interpreted through symptoms, neurological status, dynamic stability, cord and foraminal reserve, recurrence, and sport-specific exposure. Congenital variants should be distinguished from acquired or sport-adapted morphology, and a single radiographic metric should not determine eligibility. Most recommendations remain constrained by heterogeneous literature and a scarcity of prospective athlete-specific studies.

Accordingly, the most defensible approach is individualized and multidisciplinary: define the finding precisely, select imaging according to mechanism and symptoms, identify red flags, document the evidence basis, and use shared return-to-play decisions with follow-up when recurrence or progression is possible. Prospective registries and standardized anatomical reporting are required before variant-specific risk estimates or universally applicable contraindications can be established.

## Figures and Tables

**Table 1:** Cervical variants and morphologies: mechanisms, clinical concerns, and evidence basis.

Evidence Basis and Key References	Typical Sport Context	Principal Clinical Concern	Sport-Relevant Mechanism	Principal Level	Variant/Morphology
Athlete cohorts and reviews [8,9,23,24,25,29,30,32]	Football, rugby, hockey, wrestling, and other collision sports	Cervical cord neurapraxia, transient quadriparesis, and reduced neurological reserve	Smaller canal and CSF buffer during flexion-extension or axial load	Usually C3–C7; often multilevel	Developmental canal stenosis
Reviews and expert guidance [31,32,33,41,42,43]	Collision/contact sports and forceful trauma	Recurrent bilateral symptoms, potential myelopathy, and return-to-play restriction	Dynamic cord compromise from congenital and acquired narrowing	Any cervical level; usually subaxial	Functional stenosis
Athlete cohorts and clinical reviews [10,11,12,34,35]	Football, rugby, hockey, and wrestling	Stingers, burners, and radiculopathy	Root or plexus traction and foraminal compression during extension, rotation, or lateral bending	Usually C4–C7	Foraminal narrowing or disc-height loss
Case reports [15,16,38]	Football and other trauma sports	Diagnostic confusion with fracture; instability or cord risk in selected configurations	Ring defect may mimic fracture; consequences depend on stability and associated anomalies	C1	Atlas arch defect or bipartite atlas
Case report and clinical guidance [17,44,45,46,47]	Boxing, adaptive sport, and contact exposure	Atlantoaxial instability, myelopathy, and cord injury	Odontoid abnormality may permit dynamic C1–C2 translation	C2	Os odontoideum or odontoid anomaly
Reviews, expert guidance, and case literature [36,37,38,48,49]	Contact and collision sports	Adjacent-segment disease, transient neurological symptoms, and clearance uncertainty	Fusion redistributes motion and may coexist with upper-cervical abnormalities	Any level; greater concern when upper cervical or multilevel	Klippel–Feil syndrome or congenital fusion
Case reports; one biomechanical analysis [13,18,50]	Judo, football, and baseball	Neck pain, radiculopathy, and spondylolisthesis	Posterior-element defect may increase motion and disc stress, especially in rotation	Most often reported at C6	Cervical spondylolysis
Meta-analysis, systematic review, athlete cohorts, and case report [14,26,27,28,39,40,51]	Overhead, throwing, swimming, endurance, and strength sports	Neurogenic, venous, or arterial TOS	Reduced outlet dimensions may become symptomatic with repetitive loading or hypertrophy	C7/cervicothoracic junction	Cervical rib or elongated transverse process

Evidence categories are descriptive. Case reports identify possible mechanisms or management issues but do not establish incidence, causality, or universal return-to-play rules. Abbreviations: CSF, cerebrospinal fluid; KFS, Klippel–Feil syndrome; RTP, return to play; TOS, thoracic outlet syndrome.

**Table 2:** Concise vertebra-by-vertebra map of sport-relevant cervical findings.

Selected References	Athlete-Specific Evidence	Primary Clinical/Biomechanical Issue	Principal Variants or Morphologies	Level/Region
[45,46,47,52,53]	Historical screening and policy literature; limited prospective outcome data	Brainstem or cord compression and instability during flexion-extension or falls	Occipitalization, basilar invagination, CVJ anomalies, and Down syndrome-related laxity	C0–C1
[15,16,38]	Football and trauma case reports	Differentiate congenital ring defect from fracture; assess stability and cord	Anterior or posterior arch defects, bipartite atlas, and fusion failure	C1
[17,44,46,53]	Boxing case and screening or guidance literature	Dynamic C1–C2 translation and cord risk	Os odontoideum, odontoid hypoplasia, and atlantoaxial instability	C2
[36,37,43,49]	Return-to-play guidance; limited athlete outcome data	Adjacent-segment motion and associated upper-cervical abnormalities	Congenital fusion or block vertebra	C2–C3
[8,9,23,24,25,32,54,55]	Collision-sport and pediatric relevance; usually multilevel	Reduced cord reserve and transient neurapraxia	Developmental narrowing or functional stenosis	C3–C4
[8,10,11,12,32,34]	Football, rugby, and wrestling; usually part of multilevel disease	Cord or root compression during extension-compression	Canal or foraminal narrowing and disc morphology	C4–C5
[5,12,34,56,57,58]	Water-sport injury, rugby degeneration, and collision-sport symptoms	Radiculopathy, stingers, and canal compromise	Disc degeneration, foraminal stenosis, and canal narrowing	C5–C6
[13,18,50]	Judo, football, and baseball case reports	Hypermobility, spondylolisthesis, and disc stress	Cervical spondylolysis or pars/pedicle-laminar defect	C6
[14,26,27,28,39,40,51,59,60]	Overhead and TOS literature plus lower-cervical trauma cases	Root or vascular compression and acute lower-cervical neurological injury	Cervical rib, elongated transverse process, and lower foraminal or disc pathology	C6–C7 and C7–T1

The table is intentionally concise; detailed interpretation and evidence limitations are provided in Section 3.2.1, Section 3.2.2, Section 3.2.3, Section 3.2.4, Section 3.2.5, Section 3.2.6, Section 3.2.7 and Section 3.2.8. Abbreviations: CVJ, craniovertebral junction; KFS, Klippel–Feil syndrome; TOS, thoracic outlet syndrome.

**Table 3:** Sport-specific cervical injury patterns and anatomical modifiers.

Selected References	Reported Injury Patterns	Anatomical Modifiers of Interest	Dominant Cervical Load or Mechanism	Sport Group
Rihn et al. [5]; Torg et al. [6,9,25,61]; Meyer et al. [12]; Swiatek et al. [33].	Cervical cord neurapraxia, transient quadriparesis, stingers, radiculopathy, and fractures	Developmental stenosis, reduced Torg–Pavlov ratio, functional stenosis, foraminal narrowing, C1 variants, and C6 spondylolysis	Axial load, tackling, blocking, head-down contact, traction, and extension-compression	American football
Quarrie et al. [4]; Berge et al. [57]; Brauge et al. [58]; Bohu et al. [63]; Bornholdt et al. [65]; Kawasaki et al. [66]; Fuller et al. [79]; Castinel et al. [80].	Catastrophic spinal cord injury, stingers, radiculopathy, and degenerative pain	Canal or foraminal narrowing, acquired degeneration, and cervical stenosis	Scrum collapse, tackle, ruck or maul collision, and repetitive front-row load	Rugby union/league
Morrissette et al. [67]; Tator et al. [68]; Winder et al. [69]; Metz et al. [70]; Reynen and Clancy [71].	Cord concussion, fractures, spinal injury, and immobilization concerns	Stenosis when present and equipment-related alignment issues	Board collision, checking, falls on ice, and helmet or equipment effects	Ice hockey
Place and Enzenauer [17]; Sasa et al. [18]; Rich and McCaslin [72].	Neck pain, radiculopathy, central cord syndrome, and contact-sport restriction	C6 spondylolysis, Klippel–Feil syndrome or fusion, stenosis, and upper-cervical instability	Throws, bridging, chokes, axial rotation, and opponent-applied loads	Wrestling/judo/martial arts
Place and Enzenauer [17]; Hadley et al. [44].	Case-based cervical injury involving os odontoideum	Os odontoideum and atlantoaxial instability	Head impact, rotation, and occult neck trauma	Boxing
Banerjee et al. [1,2]; Good and Nickel [56]; Hutton et al. [64].	C5 fractures, fracture-dislocation, and spinal cord injury	Stenosis or fusion may modify neurological reserve; variants are underreported	Head-first impact, shallow water, and wave or body-board forces	Diving/water sports
Tassone and Duey-Holtz [47]; Boden et al. [73]; Yau et al. [74].	Cervical fractures, ligament injury, cord injury, and head-neck trauma	Upper-cervical instability, stenosis, congenital fusion, and Down syndrome-related concerns	Tumbling, inversion, basket toss, falls, and axial load through the head	Gymnastics/cheerleading/trampoline
Garraud et al. [26]; Jiang et al. [28]; Chandra et al. [39]; Talutis et al. [40].	Neurogenic, venous, or arterial TOS and radicular mimics	Cervical rib, elongated C7 transverse process, and outlet narrowing	Repeated abduction-external rotation and scapular or clavicular motion	Overhead/throwing/swimming
Banerjee et al. [1,2]; Hutton et al. [64]; DePasse et al. [76].	Cervical fractures, spinal cord injury, and ligament injuries	Variants are usually incidental but may affect prognosis and surgical planning	Forceful fall or crash	Cycling/equestrian/ski/snowboard/motorsport
DePasse et al. [76]; Jo et al. [77]; Meron et al. [78].	Neck sprain, neurapraxia, and rare fracture or spinal cord injury	Stenosis or instability when present; variants are rarely studied	Collision, falls, headers, and awkward landings	Basketball/soccer/lacrosse
Goldberg [45]; Hankinson et al. [46]; Tassone and Duey-Holtz [47]; Taylor and Walter [52]; Tomlinson et al. [53].	Screening and restriction debates; emphasis on symptoms, neurological findings, instability, and mechanism	Atlantoaxial instability, odontoid abnormalities, CVJ variants, and Down syndrome-related laxity	Sport-specific exposure; greatest concern with marked flexion-extension, diving, gymnastics, equestrian activity, and contact	Adaptive sports/Special Olympics

Injury patterns and anatomical modifiers vary within each sport group; this table summarizes recurring themes rather than sport-specific causal estimates.

**Table 4:** Integrated return-to-play framework for cervical anatomical findings.

Evidence Basis and Key References	Possible Return-to-Play Criteria	Required Investigations	Absolute/Near-Absolute and Relative Contraindications	Anatomical Finding and Principal Risk
Case reports [15,16,38]	Asymptomatic; normal neurological examination; stable ring; no cord or ligament injury; specialist clearance	CT for cortical morphology; MRI and dynamic assessment if symptoms or instability is suspected	Absolute/near-absolute: instability, cord compression, or neurological deficit. Relative: pain, uncertain stability, or collision exposure involving substantial contact forces.	Stable C1 arch defect or bipartite atlas—misdiagnosis as fracture and occult instability
Case report and guidance [17,44,46]	Only if asymptomatic, stable, neurologically normal, and cleared by an upper-cervical specialist; collision exposure often remains inadvisable	CT and MRI; flexion-extension radiography only when safe; specialist spine assessment	Absolute/near-absolute: unstable os, myelopathy, cord signal abnormality, or cord compression. Relative: stable asymptomatic lesion in collision sport or uncertain stability.	Os odontoideum or odontoid anomaly—atlantoaxial instability and cord injury
Cohorts and morphometric studies [23,24,25,29,30]	Normal examination; no functional stenosis, cord deformation, or cord signal abnormality; informed shared decision	MRI when the ratio is reduced, symptoms are present, or sport exposure is consequential; assess SAC and cord	Absolute/near-absolute: none on ratio alone. Relative: markedly reduced SAC, loss of CSF buffer, or recurrent neurological symptoms.	Asymptomatic developmental stenosis—reduced reserve and limited specificity of the Torg–Pavlov ratio
Reviews, cohorts, and expert guidance [8,9,31,32,35,43]	Complete symptom resolution; full painless motion and strength; normal neurological examination; no instability or ongoing cord compression; specialist clearance	MRI; CT after trauma; neurological assessment; dynamic imaging when safe; document recurrence and cord signal	Absolute/near-absolute: persistent deficit, instability, cord signal abnormality, or recurrent neurapraxia with functional stenosis. Relative: a single fully resolved episode without structural pathology.	Cervical cord neurapraxia or transient quadriparesis—recurrence and permanent injury when cord pathology is present
Athlete studies and reviews [10,11,12,34,35]	Complete recovery; normal strength, motion, and neurological examination; contributing cause addressed; recurrence plan	MRI for recurrent or atypical episodes; electrodiagnostic testing if symptoms or weakness persists	Absolute/near-absolute: persistent weakness, bilateral symptoms, myelopathy, or cord pathology. Relative: recurrent unilateral episodes or foraminal stenosis.	Recurrent stingers—chronic root or plexus injury and underlying central or foraminal stenosis
Reviews and expert guidance [36,37,48,49]	Stable and asymptomatic; normal neurological examination; adequate canal; no associated anomaly carrying substantial risk; multidisciplinary shared decision	Full cervical imaging; MRI and dynamic stability assessment when indicated	Absolute/near-absolute: upper-cervical or multilevel instability, symptomatic stenosis, or neurological deficit. Relative: stable single-level fusion in collision sport.	Klippel–Feil syndrome or congenital fusion—adjacent-segment load and associated upper-cervical abnormalities
Case reports [13,18,50]	Asymptomatic; stable or healed defect; normal neurological examination; full motion and strength; sport-specific loading tolerated	CT and MRI; flexion-extension assessment after acute injury has been excluded	Absolute/near-absolute: instability, progressive slip, or neurological deficit. Relative: pain, stress reaction, uncertain healing, or rotation/contact exposure.	C6 spondylolysis—hypermobility, spondylolisthesis, disc stress, and radiculopathy
Systematic review, meta-analysis, athlete cohorts, and case report [14,26,27,28,39,40]	Symptom-free; vascular safety confirmed; adequate recovery after decompression when performed; sport demands tolerated; specialist clearance	Provocative examination; duplex, CT angiography, or MR angiography for vascular symptoms; selected electrodiagnostic testing	Absolute/near-absolute: untreated arterial or venous compromise, thrombosis, ischemia, or progressive neurological deficit. Relative: reproducible neurogenic symptoms or unresolved compression.	Cervical rib or TOS—neurovascular compression, thrombosis, ischemia, and plexopathy

“Absolute/near-absolute” and “relative” contraindications reflect convergent expert guidance and case-based literature, not validated universal clearance rules. Decisions require specialist, sport-specific, and shared clinical assessment. Abbreviations: CT, computed tomography; MRI, magnetic resonance imaging; SAC, space available for the cord; TOS, thoracic outlet syndrome.

## Data Availability

No new data were generated or analyzed in this study. The information sources, literature-identification approach, and narrative synthesis process are described in Section 2.

## Abbreviations

The following abbreviations are used in this manuscript:

Definition	Abbreviation
cervical cord neurapraxia	CCN
computed tomography	CT
craniovertebral junction	CVJ
Klippel–Feil syndrome	KFS
magnetic resonance imaging	MRI
return to play	RTP
space available for the cord	SAC
thoracic outlet syndrome	TOS
transient quadriparesis	TQ
